# Numerical simulation study on the force of overwintering foundation support structure of unsaturated seasonal permafrost under indoor experiments

**DOI:** 10.1098/rsos.240992

**Published:** 2024-11-20

**Authors:** Haotian Guo, Xinzhu Zhao, Chao Sun, Xiangqun Li, Kai Yang

**Affiliations:** ^1^ School of Geomatics and Prospecting Engineering, Jilin Jianzhu University, Jilin Changchun 130118, People's Republic of China; ^2^ Institute of Geotechnical Engineering in the Seasonal Frozen Region, Jilin Jianzhu University, Jilin Changchun 130118, People's Republic of China

**Keywords:** unsaturated soil, matric suction, shear strength, FLAC3D, deep foundation pit

## Abstract

When analysing the effect of negative temperature on overwintering pit constructions of unsaturated soil, using the mechanical parameter of saturated soil at room temperature leads to an inaccuracy in the research findings. The strength parameters are obtained through indoor experiments. The foundation pit model is created using FLAC3D numerical simulation software based on the indoor experimental data. The influence of different parameters on the stress and deformation of the overwintering deep foundation pit supporting structure is analysed. The numerical simulation results obtained are compared with the actual monitoring data. According to research, the matric suction of the silty clay in its natural state in the Changchun area is 70 kPa. As the temperature decreases, the total cohesion of the unsaturated soil increases, and the internal friction angle tends to decrease. The numerical simulation results are consistent with the actual monitoring data changes. With the excavation, the horizontal displacement of the supporting structure increases first and then decreases, reaching the maximum displacement at two-thirds of the foundation pit. Compared with room temperature, the deformation of the supporting structure is larger under a negative temperature condition. The deformation of the supporting structure simulated by the actual temperature mechanical parameters is larger than that under the condition of normal temperature mechanical parameters. The frost-heaving force increases with the overall excavation, and a surge occurs at the bottom of the pit. The frost-heaving force changes most significantly under the condition of freezing at −20°C for 30 days.

## 1. Introduction

Frozen soil is widely distributed around the world, covering about 70% of the world’s land area [1]. In recent years, the progress of human activities and the change of the ecological environment have caused global temperatures to rise year by year. Global warming has led to the gradual transformation of some areas of perpetually frozen soil into seasonal frozen soil. Seasonally frozen soil freezes in winter and thaws in summer only within a few meters below the ground surface [2]. The construction engineering problems brought by temperature changes have also increased. The deformation and failure of deep foundation pit engineering in seasonal frozen areas for high-rise buildings is particularly severe during winter. Owing to the influence of temperature change, dry-wet cycle and artificial precipitation construction, soil in nature is unsaturated soil [3]. Using simple saturated soil parameters at room temperature while designing an overwintering foundation pit supporting structure will result in significant design errors. Therefore, it is significant to consider the changes in soil parameters during the actual overwintering process for the design of the pit support structure.

With the urgent needs of engineering, in recent years, Wu *et al*. [4], Shi [5], Charles *et al*. [6], Zhang *et al*. [7], Saleh Maha [8] and Li *et al*. [9] have chosen pit engineering as the research background to systematically study unsaturated soil pits and their supporting structures in engineering construction. In practical engineering, the method of detecting frost heaving deformation on site has the problems of low efficiency and large errors of detection data. There is also a problem that the detection results are not universal owing to regional differences. Therefore, the majority of scholars use the combination of numerical simulation and experimentation to simulate the excavation of a foundation pit. Some achievements have been made in the simulation of an overwintering foundation pit and the application of unsaturated soil theory. The nonlinear module is used to compare the numerical simulation results of a deep foundation pit with or without supporting structure [10]. Dou *et al*. [11] established a finite element model of the deep foundation pit supporting structure using PLAXIS software and examined the impact of water content on the deformation of the foundation pit. Zhang *et al*. [12] analysed the actual inspection data of the deep foundation pit in the seasonal freezing area, summarized the reasons affecting the breakage of the supporting structure, and proposed recommendations for mitigating frost heaving. Cao *et al*. [13] modelled unsaturated foundation pits using PLAXIS and investigated the impact of water content on foundation pit deformation properties. Wang *et al*. [14] developed ABAQUS software using the FORTRAN language to build up the functional link between the temperature field of deep foundation pit soil and frost heaving. Ren *et al*. [15] performed numerical simulations to investigate the effects of the support forms of pile anchors, double-row piles and diagonal bracing combinations on deep foundation pits. Li *et al*. [16] created the fundamental equations for the soil temperature field and stress field by combining examples, then simulated the thermal-mechanical coupling of the foundation pit supporting structure using ANSYS finite element analysis software. Sun *et al*. [17] examined the deformation of the foundation pit and supporting structure by simulating the overwintering excavation of the pile-anchor supporting foundation pit using FLAC3D software. Wu [18] used FLAC3D to simulate overwintering for Harbin subway deep foundation pits and analysed soil displacement and freezing force, and provided anti-freezing and expansion measures. Zhang [7] established a three-dimensional foundation pit model to study the effects of water-force coupling and unsaturated soil properties on the foundation pit.

In summary, it can be seen that the existing research on foundation pits mainly focuses on the establishment and optimization of numerical models. However, for engineering construction in the seasonal frozen area, the change of temperature and the effect of saturation have a great influence on soil samples. As for soil research, unsaturated soil research focuses on positive temperature research, while frozen soil research is mostly unsaturated soil. There are relatively few studies on unsaturated frozen soil at different freezing temperatures. Therefore, it is necessary to further explore the influence of matric suction on the mechanical properties of frozen soil and apply the obtained parameters at different temperatures to engineering simulation analysis to solve practical engineering problems.

In light of the aforementioned study gaps, this paper uses silty clay in Changchun, China, a typical seasonally frozen area, as the research object and conducts a soil–water characteristic experiment to measure the matric suction of unsaturated soil under natural water content. Then, triaxial shear tests were conducted on natural unsaturated soil and saturated soil at different temperatures to study the effect of overwintering temperature changes on the strength parameters of unsaturated soil. The excavation of an overwintering foundation pit is simulated using FLAC3D numerical simulation software based on the indoor experimental data. The numerical simulation results are compared with the measured data. The force and deformation law of an overwintering deep foundation pit is analysed by different mechanical parameter selections. The study’s findings are anticipated to offer theoretical support and direction for overwintering foundation pit engineering design and construction.

## 2. Source and properties of soil samples

### 2.1. Source of soil samples

**Figure 1.**
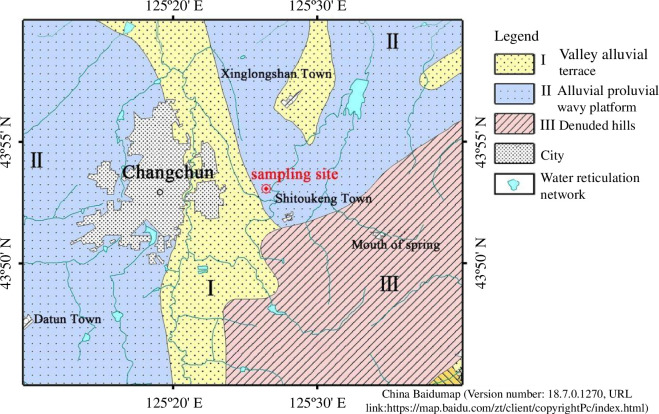
Engineering geological landform sketch of the sampling site.

Soil samples were taken from Changchun City, Jilin Province, near the East Huancheng Road station of rail transit line 7. The sampling site is located in the geomorphic unit of the alluvial terrace (I) in the Yitong River Valley, and the cover layer is mainly the quaternary alluvial clay layer and sand layer. Figure 1 depicts the engineering geological landform of the sampling site. The groundwater depth was 2.5–3.1 m during the survey period, and atmospheric precipitation and surface water infiltration were the main sources of groundwater replenishment. The freezing depth ranges from 1.5 to 1.8 m. The geological lithology and distribution characteristics are listed in table 1.

**Table 1.** Geological lithology and distribution characteristics.

no.	designation	soil thickness	distribution characteristics
1	Q4 ml	0.8–1.6	the artificial accumulation layer is mainly greyish black or yellowish brown, slightly wet and loose. It is mainly composed of cohesive soil, including plant roots and a small amount of gravel. The layer is continuously distributed
2	Q4 al	5.2–7.2	the Quaternary Holocene alluvial layer is mainly yellowish brown in colour. Plastic state, local plasticity. Iron-manganese nodules, no shaking reaction, slightly glossy. The dry strength is medium, toughness is medium and compressibility is medium. The layer is continuously distributed
3	Q4 al	4.4–9.7	the Quaternary Holocene alluvial layer is mainly brownish yellow. Local plastic state, no shaking reaction. Slightly glossy, medium dry strength, medium toughness, medium compressibility. The layer is continuously distributed
4	Q4 al	3.4–8.4	the Quaternary Holocene alluvial layer is mainly brownish yellow. Hard plastic state, no vibration response. Slightly glossy, medium dry strength, medium toughness, medium compressibility. The layer is continuously distributed
5	K1	1.9–3.9	the Cretaceous mudstone is mainly reddish brown and grey. It is in fully weathered condition, with muddy structure and laminated structure. The structure is basically destroyed, with residual structural strength. Hammering sound dumb, no rebound, hand breaking fragile. It is easy to soften in water and disintegrates in water loss. Dry drilling can be drilled, and the rock mass is extremely broken, which belongs to extremely soft rock. The basic quality grade of the rock mass is grade V, and the layer is continuously distributed

Geological drilling was performed in this exploration using the XY-150 and DPP-100 engineering drilling rigs. The diameter of the finished hole was 108 mm, with an opening diameter of 146 mm. Soil sampling, transportation and preservation of soil are in accordance with ASTM standards [19,20]. The silty clay at 4–5 m below the freezing depth was chosen as the research object in this experiment to prevent the impact of freeze-thaw cycles on soil properties in winter.

### 2.2. Particle size and basic physical properties of soil samples

**Figure 2.**
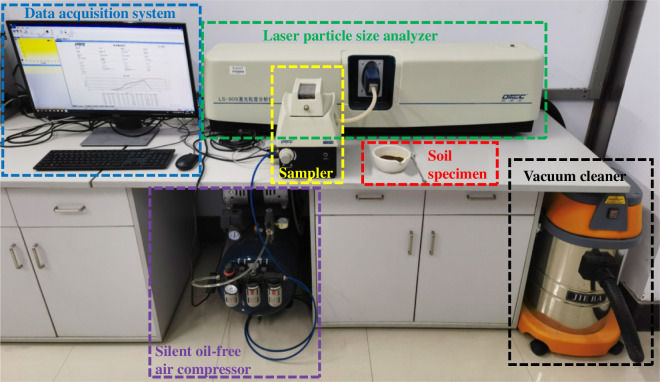
Laser particle size analyser (BT-9300H).

To ascertain the relative content of each particle size in soil samples and to clarify the features of soil samples, particle analysis tests on 12 groups of silty clay were carried out using a laser particle size analyser (BT-9300H), as shown in figure 2.

**Figure 3.**
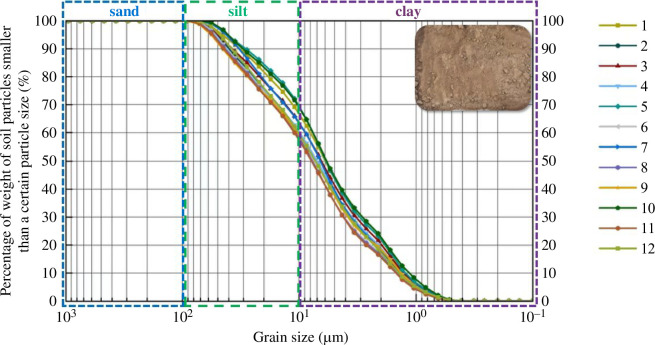
Particle size distribution of the soil.

Each group took a suitable amount of soil, which was dried for 8 h in an oven at a temperature between 105 and 110℃. After drying, the soil sample was ground into powder and put into a laser particle size analyser for examination of particle size. Each batch of soil samples was exposed to three experiments, with the average value being used as the final result to eliminate contingencies and minimize errors. After processing and analysing the data from the device, the particle grading curves of 12 sets of soil samples were plotted, as shown in figure 3.

The calculation results of the non-uniformity coefficient are above 5, and the curvature coefficients of 12 groups are between 1 and 3 for this silty clay. The overall powder particle content accounts for more than half with good particle gradation. The soil’s internal capillary phenomenon is clear. The physical and mechanical characteristics of the soil visibly change when the temperature is negative or the water content fluctuates.

**Figure 4.**
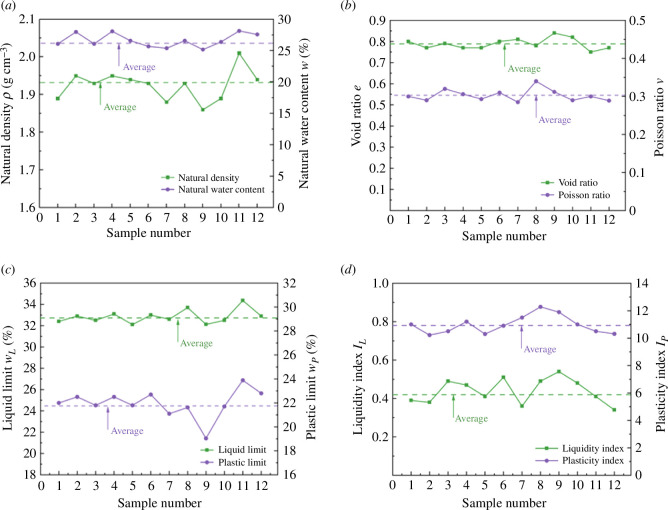
Physical and mechanical parameters. (*a*) ρ and W. (*b*) e and v. (*c*) wL and wp. (*d*) IL and Ip.

Clarifying the basic physical and mechanical properties of the sample plays an important role in analysing the mechanism of temperature change on the stress–strain curve and shear strength of unsaturated silty clay. Indoor tests were carried out according to ASTM standards [21–23] to obtain the basic physical parameters of the soil. The physical and mechanical parameters of 12 groups of soil samples obtained from the test are shown in figure 4. The range and average value are shown in table 2. The analysis shows that the water content of the soil is mainly affected by the particle composition. When the percentage of powder particles is higher, the surface area of the soil particles is larger, and the ability of the surface to adsorb water is stronger. Therefore, the liquid plastic limit of the soil also increases.

**Table 2.** Range and average of basic physical and mechanical parameters of the specimens.

physical and mechanical parameters	range	average
natural density *ρ* (g cm^−3^)	1.86–2.01	1.93
natural water content *w* (%)	25.19–28.16	26.61
void ratio *e*	0.73–0.84	0.79
Poisson ratio *v*	0.297–0.306	0.302
liquid limit *w_L_* (%)	32.12–34.36	32.9
plastic limit *w_P_* (%)	19.04–23.89	21.85
liquidity index *I_L_*	0.34–0.54	0.43
plasticity index *I_P_*	10.22–12.28	10.95

## 3. Laboratory test and scheme

### 3.1. Soil‒water characteristic test

**Figure 5.**
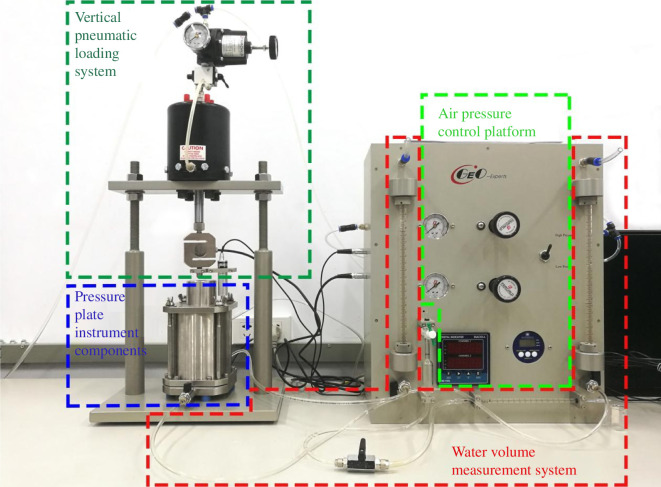
GEO-Experts soil‒water characteristic curve pressure plate instrument system.

The pore water pressure and pore pressure in unsaturated soil are not equal, so there is matrix suction inside the soil. The matric suction of the silty clay in its natural state was obtained by soil water characterization tests. A GEO-Experts pressure plate instrument was used for this experiment, as shown in figure 5. The clay plate in the instrument can act as a diaphragm between air and water. By measuring the moisture absorption curve or dehumidification curve of the soil under different stress states, the residual moisture of the soil sample after applying air pressure balance at each level is determined.

Unsaturated soil is created during the dehumidification process as a result of the influence of temperature, groundwater and other elements. This experiment saturates the soil sample with a vacuum saturation apparatus in an effort to mimic the real world as closely as possible and then the matric suction was applied step by step. There are 18 levels in the matric suction application range, which is 0–400 kPa. The matching drainage volume and volumetric water content at each matric suction were measured, the soil–water characteristic curve was constructed, and the matric suction of soil samples at natural moisture content was obtained.

### 3.2. Triaxial test

**Figure 6.**
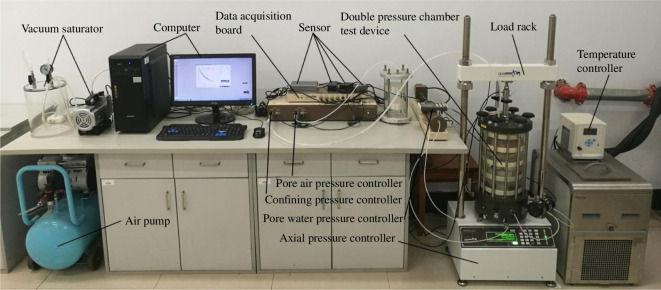
GDS unsaturated soil triaxial test system.

To study the ability of natural state (unsaturated state) soil to resist deformation breakage under different negative temperature conditions, a Geotechnical Data Systems unsaturated soil triaxial test system was used to control the matric suction of the sample and conduct the strength test under various negative temperature conditions. The test equipment, shown in figure 6, consists of three parts: the controller, the pressure chamber and the data acquisition system. The matric suction of the soil was varied by controlling the pore air pressure and counterpressure, while axial and radial pressures were applied to the soil samples to simulate the strength parameters of the soil at different negative temperatures.

**Figure 7.**
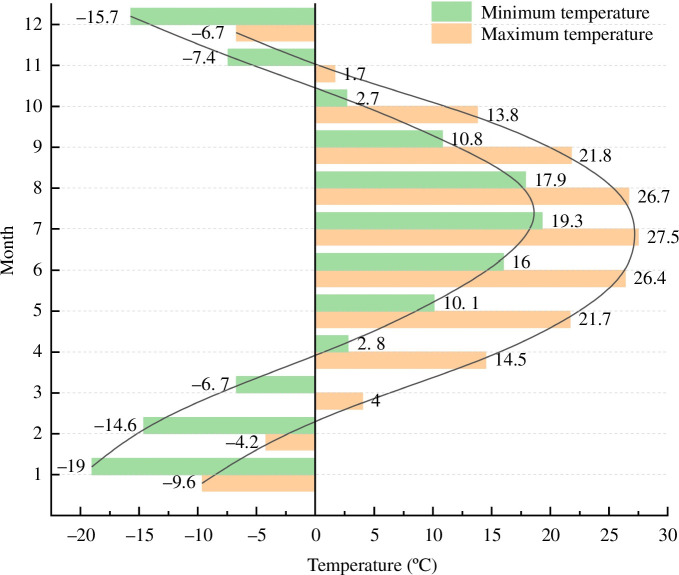
Annual temperature curve of Changchun in 2023.

In this test, the matrix suction was fixed to 70 kPa under the natural water content, and the confining pressure was set to 100, 200 and 300 kPa using the consolidated undrained triaxial test. The experimental temperatures were set to 25, 0, −2, −6, −10 and −20°C, the freezing time was 12 h according to the annual temperature profile of Changchun in 2023 in figure 7. The strain control loading method was used in the test, and the shear rate was set to be 0.1% min^-1^. The test was stopped when the strain reached 15%. The particular experimental plan is presented in table 3.

**Table 3.** Triaxial test parameter setting of silty clay.

matric suction (kPa）	temperature (°C）	net confining pressure (kPa)	pore gas pressure (kPa)	pore water pressure (kPa)
70	25, 0, –2, –6, –10, −20	100, 200, 300	75	5
0			0	0

To form a comparison with the unsaturated clay shear strengthmass water content. The saturated soil body was used for the experiment, and the variable control was consistent with the above test. The test programme is shown in table 3.

## 4. Laboratory test results and analysis

### 4.1. Analysis of soil‒water characteristic test results

**Figure 8.**
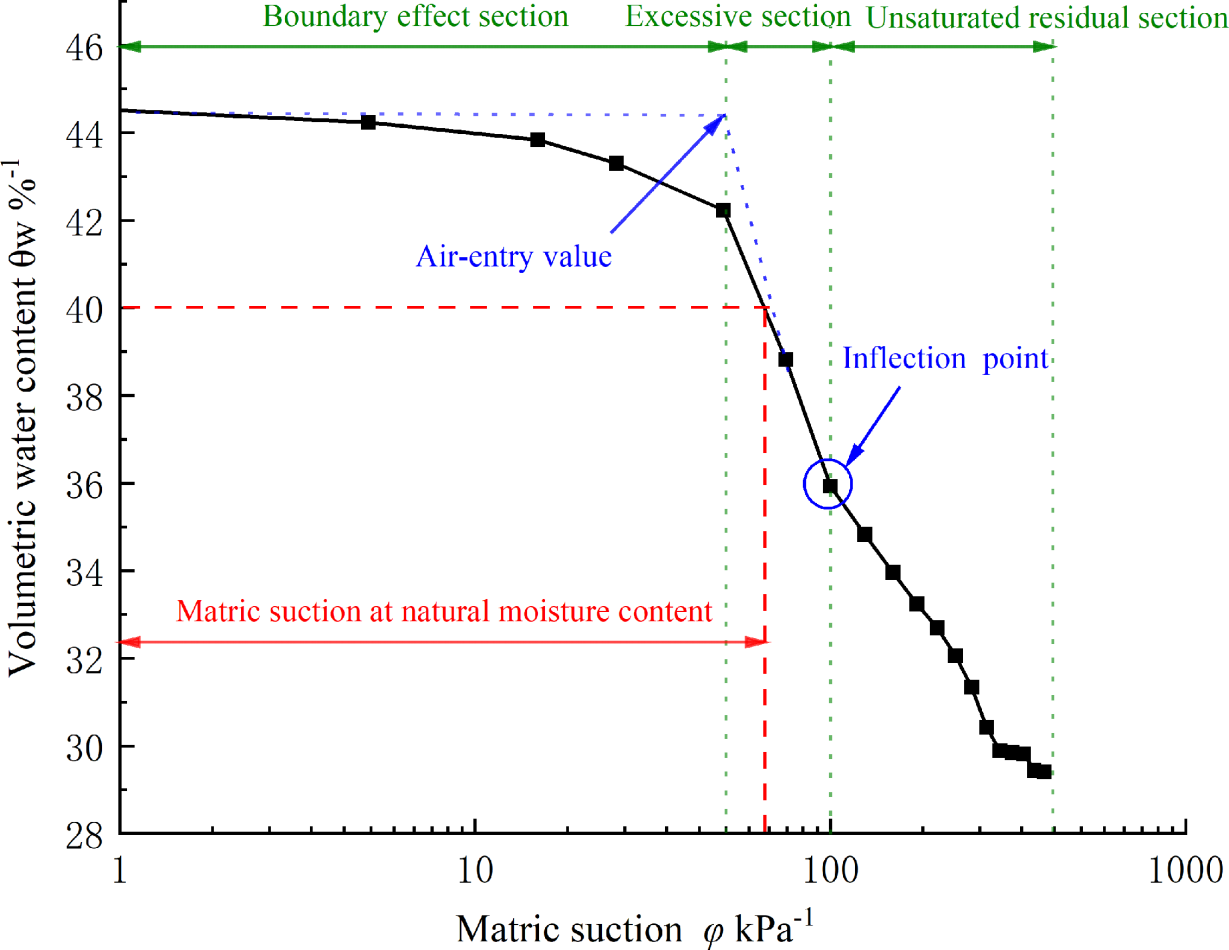
Relationship between volumetric water content and matric suction.

The soil‒water characteristic curve reflects the constitutive relationship between matric suction and water content, which can use both mass water content and volumetric water content. In this article, volumetric water content is used to represent mass water content, and the conversion relationship between the mass water content and the volumetric water content is shown in equation (4.1). The experiment’s matric suction was increased from 5 to 400 kPa, and the corresponding drainage volume and volumetric water content were measured. Figure 8 depicts the relationship curve between matric suction and volumetric water content at room temperature:

(4.1) $$\theta_{w}=\frac{S\text{r}\cdot e}{1+e}=\frac{G_{s}\cdot w}{1+e},$$

where *θ_w_* is the volumetric water content of the soil sample, *w* is the natural water content, *G_s_* is the specific gravity and *e* is the void ratio.

The volumetric water content of the soil in the natural state is calculated to be 40.58%. The matric suction of the soil in this area is approximately 70 kPa, as determined by the soil–water characteristic curve.

According to the soil–water characteristic test data, the volumetric water content of soil samples decreases with the increase of matric suction, indicating an overall negative correlation between the two. The air-enty value is obtained when the matric suction reaches 50 kPa, and it can be said that the matric suction is saturated in the region of 0–50 kPa. At this stage, the pores of the soil particles are almost filled with water, and the pore water pressure is close to the pore pressure. Although part of the water in the soil is discharged, the soil can still approximately be considered to be saturated.

The soil is in an excessive section when the matric suction is between 50 and 100 kPa. The water content of the soil decreases rapidly with the increase of the matric suction. When the matric suction reaches the air-enty value, the pores are connected with each other. A significant volume of gravity water is released from the soil pores under the impact of pressure differential and is replenished by capillary water. The matrix suction increases the adhesion force between particles. At this stage, the majority of the pore water is weakly bound water, which significantly enhances the capillary action between particles. In practical engineering, unsaturated soil is primarily observed at this stage. The soil is in the unsaturated residual stage when the matric suction is from 100 to 400 kPa, and the rate of decrease of volumetric water content slows down with the increase of matric suction. In this stage, capillary water in the pores of the soil body is gradually eliminated and finally exists in the form of strongly and weakly bound water. The pores inside the soil are almost all gas and finally tend to be stable.

### 4.2. Analysis of temperature-controlled unsaturated triaxial test results

**Figure 9.**
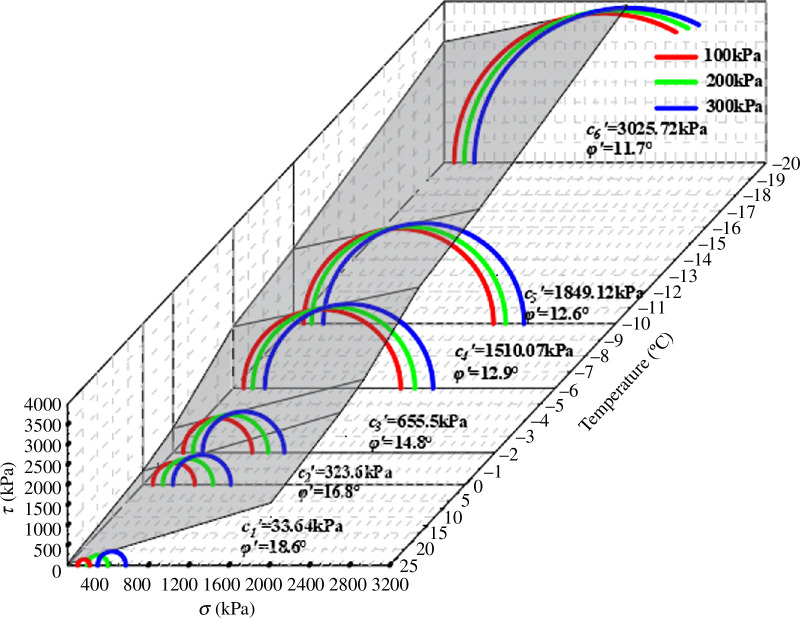
Mohr‒Coulomb stress circle and strength envelope under 70 kPa matric suction.

Using the GDS unsaturated soil triaxial test system, the triaxial shear test of unsaturated silty clay in the Changchun area with natural water content was conducted at various temperatures, and analyse the change rule of its strength index under the action of different negative temperatures. The shear strength curve of the soil was plotted based on the test data, as shown in figure 9. The angle between the shear strength curve and the horizontal coordinate is the internal friction angle of the soil, and the intercept with the vertical coordinate is the cohesion of the soil.

According to the shear strength curve, when the matric suction is 70 kPa, the slope of the straight line increases as the temperature decreases, although the shift is not immediately apparent, and the intercept between the straight line and the ordinate clearly increases. It can be concluded that the temperature has a significant impact on the soil’s cohesiveness but only a minor impact on the internal friction angle.

**Figure 10.**
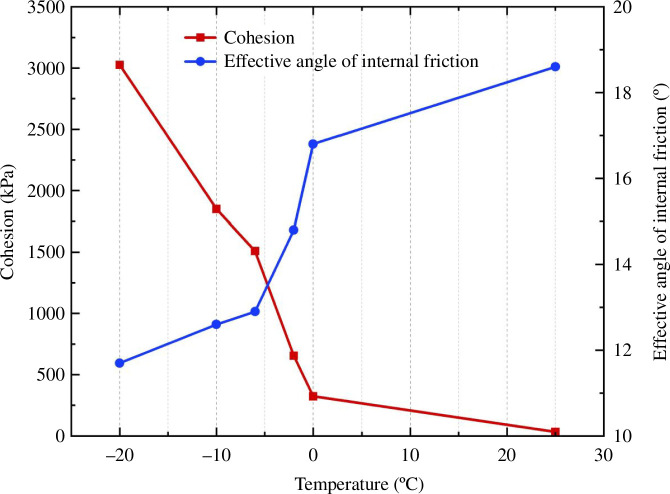
Shear strength parameter under 70 kPa matric suction.

It is clear from the cohesion change curve in figure 10 that cohesion rises as temperature falls. The negative temperature has a much larger influence than the positive temperature. The total cohesion increases by nearly 10 times when the temperature is lowered from 25 to 0 °C. The cohesion increases from 323.6 to 3025.72 kPa when the temperature drops from 0 to−20 °C. After −6°C, the amount of water in the soil freezes into ice as the temperature drops further, slowly increasing the overall cohesion.

It is evident from the effective internal friction angle curve in figure 10 that the internal friction angle rises as the temperature rises, with the negative temperature having a stronger effect than the positive temperature. The change in the internal friction angle is small when the temperature is positive. The effective internal friction angle decreases the most when the temperature is between 0 and −6°C. When the temperature is lower than −6°C, the effective internal friction angle decreases slowly.

When the soil is at a positive temperature, the water in the soil still exists as liquid water, making a change less noticeable. However, when the temperature drops from 0 to−6 °C, a significant portion of the unfrozen water in the soil turns to ice, causing the largest change range. When the temperature drops below −6°C, a significant portion of the soil’s water has turned to ice, leaving only a small amount of free water. The ice content slowly rises, reducing the range of variation for the internal friction angle and cohesiveness.

### 4.3. Analysis of temperature-controlled saturated triaxial test results

**Figure 11.**
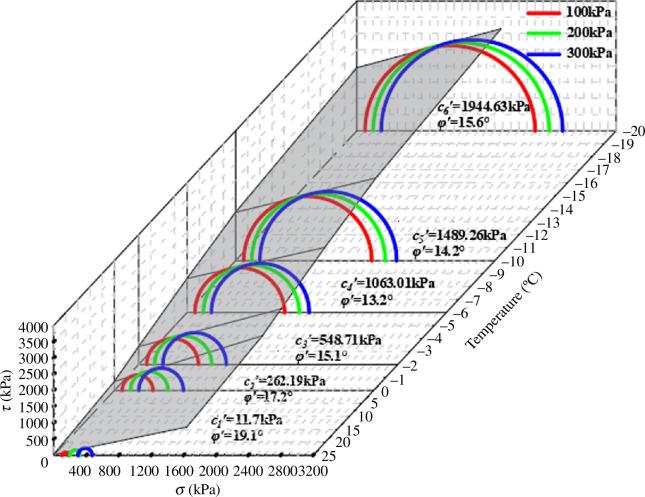
Mohr‒Coulomb stress circle and strength envelope under 0 kPa matric suction.

The triaxial shear test of saturated silty clay at different temperatures was carried out by using the GDS unsaturated soil triaxial test system. The shear strength curves are plotted by the values of cohesion and internal friction angle for different temperature conditions, as shown in figure 11.

From the shear strength curves, it can be seen that as the temperature decreases, the slope of the shear strength line decreases but the overall change is not significant, and the intercept increases significantly. It can be seen that the temperature has a relatively large effect on the cohesion of soil and a small effect on the internal friction angle.

**Figure 12.**
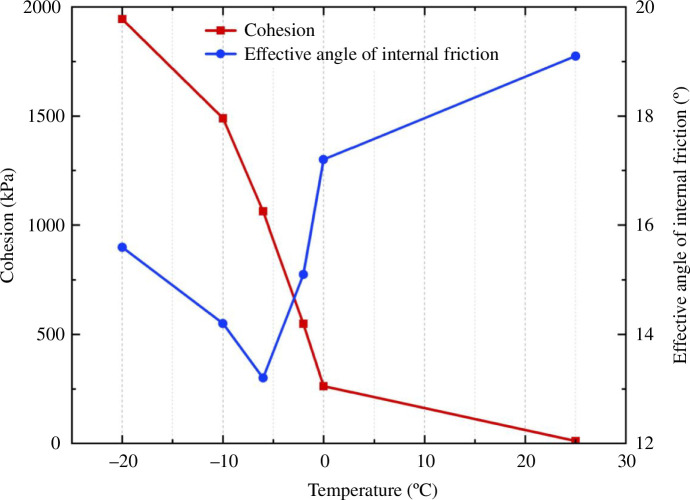
Shear strength parameter under 0 kPa matric suction.

It is evident from the cohesion change curve in figure 12 that cohesion rises as temperature falls and that negative temperature has a much larger influence than positive temperature. The cohesion increases by nearly 22 times as the temperature drops from 25 to 0°C. The cohesion increased from 262.19 to 1944.63 kPa, and the development rate somewhat decreased when the temperature dropped from 0 to−20 °C.

It is clear from the effective angle of the internal friction curve in figure 12 that when the temperature falls, the effective angle of internal friction initially decreases and subsequently increases. The internal friction angle decreases, but the amplitude does not vary significantly as the temperature drops from 25 to 0 °C. The internal friction angle continues to decrease, and the change range is wide when the temperature drops further to 6°C. The effective angle of internal friction rises from 13.2 to 15.6° as the temperature falls to −20°C.

The total cohesion of saturated soil is composed of the internal friction resistance between soil particles and the ice cementation cohesion formed by cement and water. With the decrease in temperature, the water inside the soil freezes into ice, the ice cementation cohesion increases and the total cohesion increases. The effective internal friction angle is composed of the sliding friction angle between soil particles and the static friction angle owing to particle interlocking.

When the temperature is above freezing, the lubricating effect between soil particles increases as the temperature decreases, which leads to a reduction in the effective angle of internal friction. At a temperature of 0℃, the phase change from water to ice occurs. The lubrication of ice reduces the embedding and interlocking between soil particles, decreasing the sliding frictional resistance between them. The sliding friction angle and the static friction angle between particles decrease, leading to a reduction in the total internal friction angle. An inflection point occurs when the temperature reaches −6℃. A significant portion of the water within the soil freezes into ice, leaving only a small amount of free water present. The increase in ice content slowed down significantly with the decrease in temperature. At this time, the lubrication effect of ice is reduced, leading to an increase in both the sliding friction angle and the static friction angle. Thus, saturated soil’s effective internal friction angle drops from 0 to −6 ℃ and then somewhat increases.

### 4.4. Effect of temperature on the shear strength of silty clay

**Figure 13.**
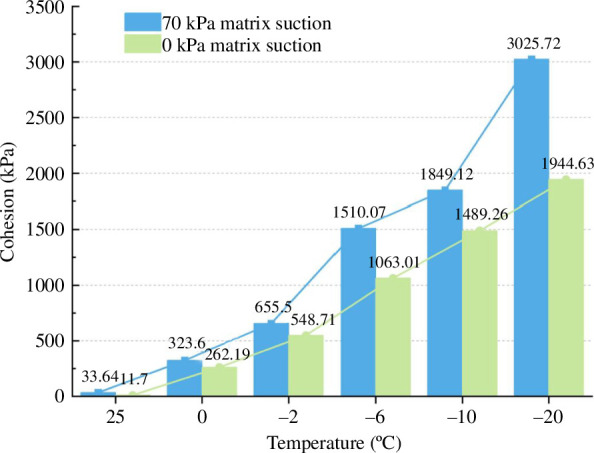
Comparison of cohesion.

**Figure 14.**
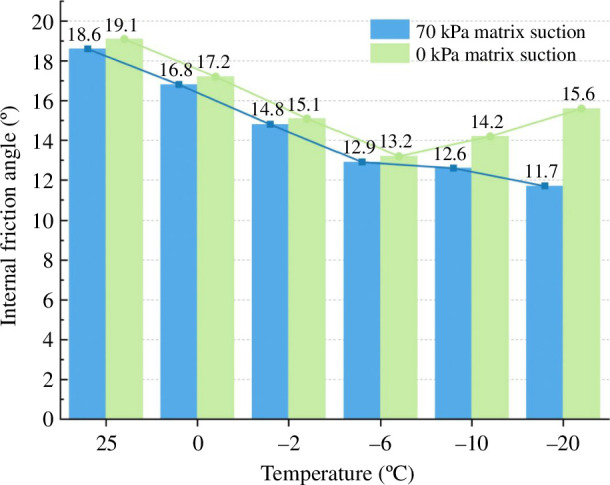
Comparison of the effective internal friction angle.

The two groups of experiments discussed above investigated the fluctuation of silty clay strength indices with matric suctions of 70 and 0 kPa at various negative temperatures. Table 4 contains a compilation of the experimental data, and figures 13 and 14 are drawn for easy observation.

**Table 4.** Triaxial test results of unsaturated silty clay and saturated silty clay.

temperature (℃)	70 kPa matric suction		0 kPa matric suction	
	cohesion (kPa)	effective angle of internal friction (°)	cohesion (kPa)	effective angle of internal friction (°)
25	33.64	18.60	11.70	19.10
0	323.60	16.80	262.19	17.20
−2	655.50	14.80	548.71	15.10
−6	1510.07	12.90	1063.01	13.20
−10	1849.13	12.60	1489.26	14.20
−20	3025.72	11.70	1944.63	15.60

The fluctuation law of the strength index in the two circumstances is essentially the same based on the chart. The cohesion rises as the temperature falls. A turning point is reached at −6°C, after which the growth tendency gradually slows. The cohesion at the same temperature is higher than that under saturated conditions because of the apparent cohesion generated by the matrix suction of the soil at the natural water content. The effective internal friction angle of unsaturated soil decreases as the temperature drops. However, the effective internal friction angle of saturated soil decreases initially before rising as the temperature drops.

## 5. Numerical simulation of an overwintering deep foundation pit based on laboratory test parameters

When digging and building deep foundation pits in seasonally frozen places, the impact of temperature must be completely taken into account. The soil’s free water starts to freeze once it reaches the initial freezing point when the winter comes. More water turns into ice as the temperature progressively drops, which causes the soil to expand. The expansion of the soil causes excessive displacement of the pit and deformation of the supporting structure, and damage to the pit occurs. Based on the indoor test settings, FLAC3D numerical simulation software is used to simulate the excavation of the foundation pit to investigate the impact of various mechanical parameters on the stress and deformation law of the overwintering deep foundation pit. The results of the numerical simulation are compared with the actual monitoring data. The study’s findings serve as a guide for overwintering foundation pit engineering in a permanently frozen region.

### 5.1. Field monitoring results

**Figure 15.**
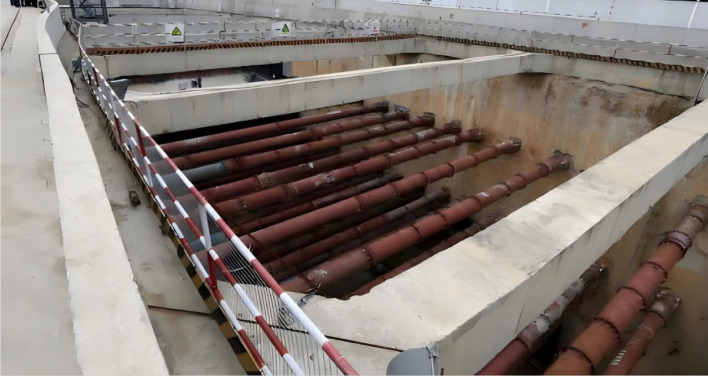
Overwintering deep foundation pit construction site.

The design and construction of a foundation pit and supporting structure were carried out in a building site in Changchun, and the results were compared with the numerical simulation results. The construction site of the overwintering deep foundation pit is shown in figure 15. In order to analyse the frost-heaving characteristics of the overwintering foundation pit, the following monitoring items are set up: surface temperature monitoring, horizontal displacement of supporting pile, and frost-heaving force of pile side. The equipment and instruments used in the monitoring project during the overwintering deep foundation pit are shown in table 5.

**Table 5.** Monitoring items using equipment and instruments.

monitoring items	equipment and instruments	model
surface temperature	intelligent temperature sensor	YH61-A08
horizontal displacement of supporting pile	total station	Ts02PLUS
frost-heaving force	vibrating wire soil pressure transducer	YH03-G10

This experiment recorded the results after freezing for 30 days in January, as shown in table 6. The actual monitoring data is compared with the numerical simulation results to verify the accuracy of the numerical simulation.

**Table 6.** Measured data of the overwintering deep foundation pits.

minimum temperature (°C）	maximum temperature (°C）	maximum horizontal displacement of supporting pile (mm）	maximum frost-heaving force (kPa）
−19	−9.6	17	2210

### 5.2. Selection of the constitutive model

FLAC3D incorporates a variety of material models, and each model is developed to correlate to a specific type of constitutive characteristics of geotechnical materials. The model is numerically simulated using the Mohr–Coulomb model. The Mohr–Coulomb model is suitable for shear–stress yield and can be well applied to geotechnical mechanics analysis such as slope stability and underground excavation. This model uses the Mohr–Coulomb strength envelope as the yield surface and includes only one failure plane, making it an idealized elastic-plastic model. The bulk modulus (*K*) and shear modulus (*G*) are used in FLAC3D to measure the material properties. The Mohr–Coulomb model requires only a few parameters of elastic modulus (*E*), Poisson ratio (*ν*), cohesion (*c*) and angle of internal friction (*φ*) to obtain a more accurate simulation. The expression formula is shown in equation (5.1):


(5.1) $$K=\frac{E}{3\left(1-2\nu \right)},G=\frac{E}{2\left(1+\nu \right)}.$$

### 5.3. Model conditions and model establishment

**Figure 16.**
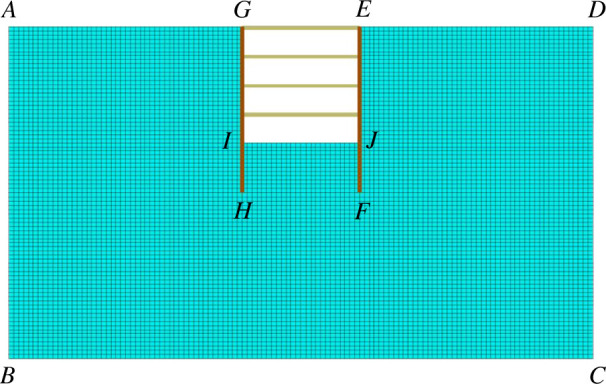
Excavation diagram of the foundation pit.

The FLAC3D two-dimensional modelling used for the foundation pit modelling simulates excavation in the ranges of 0–60 m, 0–1 m and 0–34 m in the *x*, *y* and *z*-axes, respectively. The foundation pit is 12 m long, 1 m wide and 12 m deep. Supported by supporting piles and internal supports, the piles are 17 m in length and the bottom of the piles are embedded in the soil for 5 m. Figure 16 depicts the foundation pit excavation model. Two temperature states—the normal temperature state of 25°C and the freezing state that is frozen for 30 days under conditions of −20°C—are set for the influence of the mode temperature on foundation pit excavation. The working conditions are formulated in accordance with the actual situation, as shown in table 7.

**Table 7.** Construction conditions of foundation pit.

working condition	construction content	remark
1	initial stress field	
2	applying supporting structure	
3	excavation of the first soil layer. Set the first internal support	excavation to −3.0 m elevation
4	excavation of the second soil layer. Set the second internal support	excavation to −6.0 m elevation
5	excavation of the third soil layer. Set the third internal support	excavation to −9.0 m elevation
6	excavation of the fourth soil layer. Set the fourth internal support	excavation to −12.0 m elevation
7	pit freezing at −20°C for 30 d	mechanical parameters at room temperature
8	pit freezing at −20°C for 30 d	mechanical parameters of soil temperature in different depths

### 5.4. Establishment of boundary conditions

In numerical analysis, the model will slip or twist if the displacement constraint is not applied to the model. In this simulation, the horizontal directions of AB and CD edges are constrained, and the horizontal and vertical directions of BC edges are constrained so that no displacement occurs. The boundary of the top surface of the model is free to conform to the actual deformation.

This simulation does not consider water migration, assuming that the groundwater level is constant and there is no atmospheric precipitation. It is assumed that the ground elevation is ±0 m after the site is levelled. The groundwater level is located 1 m below the bottom of the deep foundation pit after precipitation.

Set the temperature boundary conditions of the model according to the actual situation. The front, back, left, right, and bottom surfaces of the model are set as insulating boundaries. The initial temperature of the ground layer is defined as 5°C, and a temperature field of −20°C is applied to the AG, GI, IJ, EJ and ED edges.

### 5.5. Selection of model parameters

To ensure that each part of the structure has the physical properties of the actual material, appropriate material properties are assigned to the structure and soil. The thermodynamic parameters of the materials are selected according to ASTM standards [24], as shown in table 8. The physical and mechanical properties of silty clay are obtained by laboratory tests, as shown in table 9. The supporting structure parameters are shown in table 10.

**Table 8.** Thermodynamic parameters of the materials.

classification	matric suction (kPa)	thermal conductivity (W•m℃)	thermal expansion coefficient (1/℃)	specific heat capacity (J•kg℃)
pile	-	1.74	-	970
inner support	-	1.74	-	970
silty clay	70	1.44	1.64 × 10^−3^	1620
	0	1.62	1.88 × 10^−3^	1780

**Table 9.** Physical and mechanical parameters of silty clay.

matric suction (kPa)	temperature (°C)	angle of internal friction (°)	cohesion (kPa)	unit weight (kPa)	shear modulus (kPa)	volume modulus (kPa)
70	25	18.6	33.64	1930	3.25E×10^6^	7.05E×10^6^
	0	16.8	323.60	1930	1.30E×10^7^	2.82E×10^7^
	−10	12.6	1849.12	1930	5.05E×10^8^	1.09E×10^8^
	−20	11.7	3025.72	1930	7.07E×10^8^	1.53E×10^8^

**Table 10.** Material parameters of the supporting structure.

classification	model	size (mm)	material	elastic modulus (GPa)	Poisson ratio
inner support	C30	400×600	reinforced concrete	30	0.2
pile	C30	Φ1000	reinforced concrete	30	0.2

### 5.6. Simulation result analysis

The temperature was initially adjusted to 5°C and subsequently frozen at −20°C for 30 days to imitate the overwintering of the foundation pit to evaluate the impact of the temperature field on the pit. After overwintering, the soil’s surface temperature is −20°C, conforming to the atmospheric temperature under actual operating conditions. The temperature of the soil steadily rises as depth increases. At 1.7 m from the bottom of the pit, the temperature reaches 0°C. Below a depth of 3.2 m, the soil temperature stabilizes at 5°C and remains unchanged. This is broadly consistent with data obtained from field trials.

**Figure 17.**
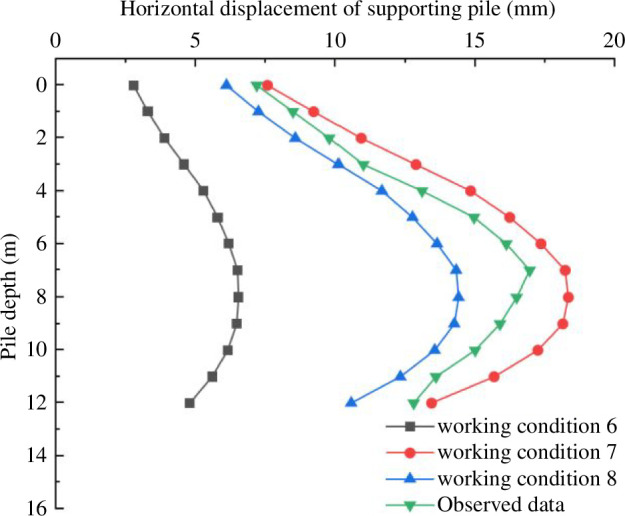
Horizontal displacement of the supporting pile.

The horizontal displacement diagram of the supporting structure is shown in figure 17. Analysis reveals the following:

- the simulation results are basically consistent with the measured data. The horizontal displacement of the supporting pile increases initially before decreasing as the foundation pit is excavated. The displacement of the pile body achieves its maximum value when the excavation depth reaches 8 m. The measured maximum displacement of the pile appears at 7 m. After that, the pile displacement starts to decrease owing to the soil pressure limitation;
- the horizontal displacement of the supporting pile under the actual engineering conditions is between the working condition 7 and the working condition 8. This result is owing to the outdoor temperature fluctuating in actual engineering scenarios, with the lowest temperature reaching −19°C. Therefore, the maximum displacement of the measured data is less than the data under the condition of −20°C constant temperature freezing. After 30 days of freezing at −20°C, the maximum displacement is significantly larger than it is at normal temperature. The maximum horizontal displacement of different layer temperature mechanical parameters is 2.21 times higher than that of the normal temperature, and the mechanical parameters of the normal temperature are 2.79 times higher than that of the normal temperature. This is brought on by the soil’s frost heaving at −20°C, which raises the earth pressure value and the horizontal displacement; and
- the horizontal displacement of the supporting pile in condition 8 is less than that in condition 7, and the actual measurement data is between the two. In comparison to the normal temperature condition, the horizontal displacement of the supporting structure is more affected by the excavation of deep foundation pits under freezing conditions. Negative temperatures induce frost heaving in the soil; parameters such as the modulus of elasticity and cohesion are greater than those of the same soil at room temperature. The elastic modulus reflects the ability of soil to resist deformation. The larger the elastic modulus is, the smaller the soil deformation is. Cohesion is one of the important indices to measure the shear strength of soil. The larger the cohesion is, the greater the shear strength of the soil is. Therefore, the horizontal displacement under negative temperature is larger than that under positive temperature.

**Figure 18.**
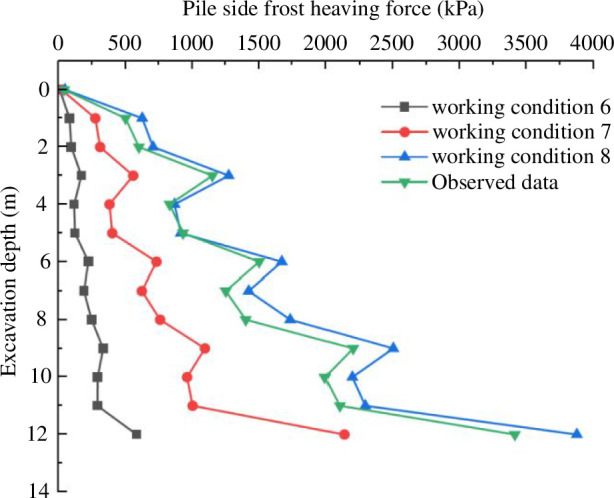
Change curve of the frost-heaving force.

The change of frost-heaving force with excavation depth under different temperature conditions is shown in figure 18. The analysis reveals the following:

- as the excavation depth increases, the freezing force on the pile side tends to increase in the actual project and three working conditions. The freezing force changes significantly in the area where the inner support is applied inside the pit. Higher lateral soil pressure is applied to the side of the pile as a result of the inner support of the foundation pit limiting the horizontal displacement of the surrounding soil. The horizontal direction restriction is relatively reduced without applying the inner support part, so the displacement becomes larger;
- at the bottom of the pit 12 m, the frost-heaving force of the pile side increases sharply under the actual project and both temperature conditions. The support structure restricts the horizontal displacement to increase the soil pressure on the pile side, and the freeze-up force on the pile side at the bottom of the pit undergoes a surge; and
- the data of the actual engineering project is consistent with the change rule of working condition 8. The actual engineering results are smaller than the numerical simulation results. This is because condition 8 imposes a constant negative temperature, which makes the frost heaving of the soil larger than the actual construction, so the frost-heaving force increases slightly. The frost-heaving force on the pile side is significantly higher after freezing at negative temperatures compared to that at normal temperatures, and the mechanical parameters of soil temperature at various depths are slightly higher. The soil’s frost heaving under conditions of negative temperature results in a higher soil pressure than under conditions of normal temperature, and the force of the frost heaving likewise rises as a result. The selection of different soil temperature parameters leads to variations in the physical properties of the soil. In condition 8, using the mechanical parameters of soil temperature at different depths results in larger bulk modulus and shear modulus, and consequently smaller horizontal displacements. Therefore, under the same freezing conditions, the frost-heaving force on the pile side is greater in condition 8.

## 6. Conclusion

The matric suction of unsaturated soil was determined by a soil water characteristic experiment. Triaxial shear tests were carried out on silty clay with different saturation levels under different negative temperatures to obtain the variation law of its strength parameters. Based on the indoor test parameters using FLAC3D, a numerical simulation study on the force deformation of an overwintering deep foundation pit support structure was carried out. The numerical simulation results are compared with the field monitoring data. These are the conclusions:

- the soil–water characteristic test shows that silty clay in the Changchun area has a matric suction of 70 kPa in its natural state. According to the analysis of the triaxial test, the strength parameters of saturated and unsaturated soil fluctuate with temperature in essentially the same ways. The cohesion of soil increases as the temperature drops, and unsaturated soil with a 70 kPa matric suction has stronger cohesion than saturated soil. The effective internal friction angle decreases as the temperature drops. The internal friction angle of saturated soil is greater than that of unsaturated soil at the same temperature;
- as the excavation depth increases, the horizontal displacement of the piles increases first and then decreases, with the maximum displacement occurring at one-third of the way from the bottom of the pit. The displacement of the supporting structure is more affected by the negative temperature. Under freezing conditions, the horizontal displacement of the supporting piles increases by 2.21 times when using the soil’s normal temperature parameters and by 2.79 times when using the soil temperature parameters at different depths, compared to the displacements at normal temperatures. It is shown that negative temperatures cause freezing and expansion of the soil, leading to an increase in soil pressure and leading to an increase in the horizontal displacement of the supporting structure;
- the frost-heaving force on the pile side increases with the increase in excavation depth. The internal support part change in the foundation pit is more visible. There is a surge phenomenon at the bottom of the pit. The effect of the negative temperature on the frost-heaving force is noticeably larger than that of the normal temperature. The frost heaving pressure simulated by the actual temperature mechanical parameters is larger than that under the condition of normal temperature mechanical parameters; and
- the results of the numerical simulation are consistent with the actual monitoring data. It shows that numerical simulation can better reflect the stress and deformation characteristics of overwintering deep foundation pits. Through modelling and analysis, it was discovered that the choice of temperature mechanical parameters for the various soil layers had an impact on the simulation outcomes. Negative temperatures had a clear impact on the foundation pit’s supporting structure. Negative temperatures induce frost heaving in the soil, leading to an increase in soil pressure. The horizontal displacement of the supporting structure and the frost-heaving force on the pile side both exhibit an increasing trend as the temperature drops. In practical engineering, choosing the appropriate temperature parameter can better imitate the real scenario. This study serves as a theoretical guide for foundation pit engineering construction.

## Data Availability

Data have been submitted as required. The analysis and results, as well as the process of data analysis and processing, can be made available by contacting the corresponding author if the editor and reviewers have questions about them.
